# The use of liposomal bupivacaine in fracture surgery: a review

**DOI:** 10.1186/s13018-023-03583-1

**Published:** 2023-04-01

**Authors:** Andrew D. Gailey, Robert F. Ostrum

**Affiliations:** 1 Department of Orthopaedic Surgery, University of Tennessee Health Science Center-Campbell Clinic and University of North Carolina Health Care, 1584 Forrest Ave, Memphis, TN 38112 USA; 2 Department of Orthopaedic Surgery, University of North Carolina at Chapel Hill, Chapel Hill, NC USA; 3 Department of Orthopaedic Surgery, Campbell Clinic/University of Tennessee Health Science Center, Memphis, TN USA

**Keywords:** Orthopedic surgery, Liposomal bupivacaine, EXPAREL, Fracture, Postoperative pain, Multimodal

## Abstract

Historically, opioids have played a major role in the treatment of postoperative pain in orthopedic surgery. A multitude of adverse events have been associated with opioid use and alternative approaches to pain relief are being investigated, with particular focus on multimodal pain management regimens. Liposomal bupivacaine (EXPAREL) is a component of some multimodal regimens. This formulation of bupivacaine encapsulates the local anesthetic into a multivesicular liposome to theoretically deliver a consistent amount of drug for up to 72 hours. Although the use of liposomal bupivacaine has been studied in many areas of orthopedics, there is little evidence evaluating its use in patients with fractures. This systematic review of the available data identified a total of eight studies evaluating the use of liposomal bupivacaine in patients with fractures. Overall, these studies demonstrated mixed results. Three studies found no difference in postoperative pain scores on postoperative days 1–4, while two studies found significantly lower pain scores on the day of surgery. Three of the studies evaluated the quantity of narcotic consumption postoperatively and failed to find a significant difference between control groups and groups treated with liposomal bupivacaine. Further, significant variability in comparison groups and study designs made interpretation of the available data difficult. Given this lack of clear evidence, there is a need for prospective, randomized clinical trials focused on fully evaluating the use of liposomal bupivacaine in fracture patients. At present, clinicians should maintain a healthy skepticism and rely on their own interpretation of the available data before widely implementing the use of liposomal bupivacaine.

## Introduction

### Background

An estimated 5.5 million orthopedic procedures were performed in the USA in 2010 [1]. With an aging population, this number continues to increase and it is projected that the incidence of hip fractures may increase to over 1 million annually by 2050 [2]. Similarly, by 2030, the demand for primary knee and hip arthroplasty procedures is expected to increase by 673 and 174%, respectively [3]. This increase in procedural volume places a heavy emphasis on improving the efficiency and success of preoperative and postoperative care to most effectively utilize limited hospital resources. Further, any inefficiency or failure in the preoperative or postoperative care settings has the potential to magnify and create major stresses in the overall delivery of health care.

Historically, opioids have been used liberally for the management of postoperative pain. In 2009, orthopedic surgeons wrote more opioid prescriptions than any other surgical specialty and accounted for 7.7% of all opioid prescriptions written in the USA. Postsurgical pain can persist for days or weeks and is linked to adverse events such as thromboembolic or pulmonary complications and the development of chronic pain. Contrarily, many studies have demonstrated an association between opioid levels and risk of postoperative complications, including higher rates of deep venous thrombosis and gastrointestinal and respiratory complications [4–6]. Opioids also carry a high risk of addiction. Therefore, a balance must be achieved between relieving postoperative pain and limiting opioid use. One major advancement toward achieving this balance has been the introduction of multimodal approaches to postoperative pain relief which are now widely accepted and recommended whenever possible [7].

#### Multimodal analgesia and liposomal bupivacaine

Multimodal analgesia approaches may include a combination of central (i.e., epidural), central regional (i.e., neuraxial), peripheral regional (peripheral nerve block and intra-articular or surgical site infiltration), and intravenous patient-controlled analgesia [8, 9]. Using a combination of agents with varying mechanisms of action allows for a lower dose of each agent to be used, decreasing the risks for toxicity and adverse events [8]. The benefits of reduced dose requirements are particularly important in regard to opioids due to their wide variety of adverse events, ranging from nausea and vomiting to respiratory depression [10]. Some studies have investigated the costs of these opioid-related adverse events (ORAEs) and noted that they increase hospital costs and length of stay in addition to impacting postsurgical milestones [9].

There are many approaches to multimodal analgesia that may effectively reduce the need for opioids. One important component of multimodal analgesia may be injection of local anesthetic into the intra-articular space or surgical site. Bupivacaine is a commonly used local anesthetic which has a very low rate of significant adverse events. Although bupivacaine has a longer-lasting duration of action compared to lidocaine, it still typically only lasts between 4 and 8 hours [11]. In 2011, liposomal bupivacaine (EXPAREL, Pacira Pharmaceuticals, San Diego, CA) was approved by the United States Food and Drug Administration (FDA) for local injection for the management of postoperative pain in patients undergoing bunionectomy and hemorrhoidectomy [12, 13]. These indications have since been expanded, in 2018 to include interscalene brachial plexus nerve blocks, and in 2021 to include local injection in surgical sites in children age 6 and older [14, 15]. This new formulation of bupivacaine incorporates DepoFoam drug delivery system (Pacira Pharmaceuticals, Inc.) to encapsulate the local anesthetic into multivesicular liposome particles (diameter, 10–30 μm). Lamellar liposome technology has existed for decades, however, multivesicular liposomes are structurally distinct in that they consist of hundreds of water-filled polyhedral compartments, separated by bi-layered lipid septa [16]. The unique microstructure of multivesicular liposomes is thought to result in more efficient drug encapsulation in addition to a more reliable and consistent drug release [16]. In clinical studies, DepoFoam has been shown to result in sustained drug release over several days to weeks after non-vascular administration [17]. It is theorized that there may be a decrease in the burden of opioid use if this DepoFoam technology is applied to the administration of bupivacaine and combined with a multimodal analgesia approach [11].

Liposomal bupivacaine has been investigated in numerous trials for the treatment of postoperative pain after total joint replacement. While some of these studies have demonstrated benefit in the form of decreased rescue opioid use, decreased total opioid use, fewer ORAEs, and shorter hospital stays [18–20], other studies have failed to demonstrate benefit over standard multimodal treatment protocols [14, 18, 21–26]. Pichler et al. reviewed over 88,000 cases of total knee arthroplasties performed with a peripheral nerve block of which approximately 21% of patients were also treated with liposomal bupivacaine intraoperatively [26]. This study failed to find any reduction in inpatient opioid use, length of stay, or cost of hospitalization. In contrast, a meta-analysis performed by Liu et al. found that patients treated with liposomal bupivacaine had lower consumption of morphine equivalents 24–72 h postoperatively and lower rates of ORAEs [18]. In addition to these studies on the use of liposomal bupivacaine in total knee arthroplasty, there is also an abundance of data, including meta-analyses and systematic reviews, on the use of liposomal bupivacaine in total hip arthroplasty and total shoulder arthroplasty [27–31]. Some of these studies, as well as many studies in the general surgery, and anesthesia literature fail to identify much, if any, difference between the use of standard bupivacaine and liposomal bupivacaine [32–34].

Although there is an abundance of literature on the use of liposomal bupivacaine in patient’s undergoing total joint replacement and other fields, there is very little evidence focusing on its use in orthopedic trauma and fracture patients. While it is more difficult to account for potential confounding variables in this population, versus patients undergoing elective procedures, there are also many unique benefits if liposomal bupivacaine is found to be an effective treatment option.

When treating pain in the fracture patient, opioids have often been the mainstay of treatment given that they are relatively inexpensive, have a rapid onset of action, and can achieve moderate pain relief in multiple areas at once [35, 36]. However, given high rates of ORAEs, addiction, and a recent increase in national attention to opioid abuse and misuse, many alternative methods of analgesia in trauma patients are now being investigated more [35].

## Materials and methods

Database searches were performed to systematically identify articles involving cases of surgical fracture treatment and the use of liposomal bupivacaine for postoperative pain management. All searches were conducted in July 2019. A PubMed search using the terms “liposomal bupivacaine, fracture” produced 11 results. An Embase database search using the terms (‘liposomal bupivacaine’/exp OR ‘liposomal bupivacaine’) AND ‘orthopedic’ produced 60 results. A Scopus database search using the terms “liposome AND bupivacaine AND orthopedics” produced 25 results. A Google Scholar search was also performed using the terms “liposomal bupivacaine AND fracture” which produced 2240 results. Given the relatively high volume of this search, the results were filtered to only contain results since 2011, when liposomal bupivacaine was approved by the FDA. This reduced the search to 1,360 results. Altogether, a total of 1,456 articles were identified by database search.

**Fig. 1:**
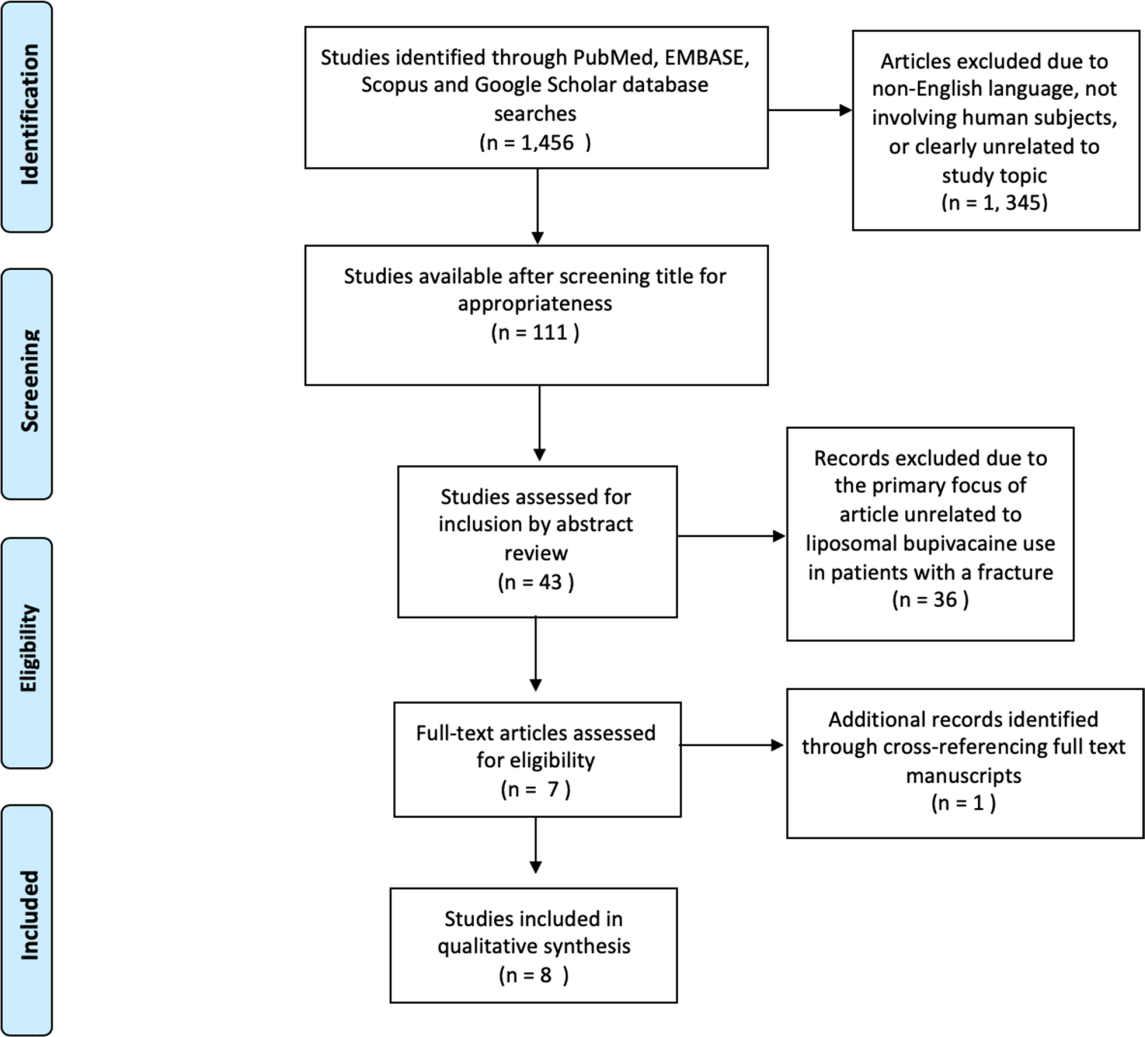
PRISMA diagram demonstrating search, screening, and exclusion processes

An initial screening process reviewed the title of each article for relevance to the study topic. All articles that were clearly not related to the study topic were excluded, this included articles in specialties other than orthopedics, as well as studies focusing on total joint replacement. Only articles written in English were included. Any animal studies were excluded. Duplicate articles were also excluded. After this initial review, there were 43 articles remaining which underwent abstract review for appropriateness. At this stage, articles were excluded if they did not have a primary focus on the efficacy or safety of liposomal bupivacaine in the setting of an injury involving a fracture. Seven studies met full inclusion criteria, and there was one additional study added after cross-referencing with the full text manuscripts (Fig. 1).

## Results

A total of eight studies were identified which met inclusion criteria and focused primarily on evaluating the use of liposomal bupivacaine in fracture patients [37–44]. The fracture type varied across studies. There were two studies on distal radius fractures, two studies on hip fractures, one study on ankle fractures, one study on isolated acetabular fractures, one case of a talar neck fracture, and a case series involving a fractured clavicle and a subtrochanteric hip fracture. The study design also varied. There were two randomized clinical trials, one prospective non-randomized trial, one retrospective review, one case series, one case report, and two expert opinion panels (Table 1).

**Table 1:** Overview of studies analyzed in this study with study characteristics and conclusions

Study	Population	Study Type	Intervention/Groups	Pain Score	Narcotic Consumption	Length of Stay	Adverse Events	Study Conclusion	Pacira Pharmaceutical sponsorship	Level of Evidence
Alter [37]	Distal radius fracture repair	Prospective, single-blinded, randomized clinical trial	Bupivacaine group: 20 mL 0.5% bupivacaine without epinephrine into incision and surgical site (*n* = 21) Liposomal bupivacaine group: 10 mL 0.5% Bupivacaine without epinephrine into incision and surgical site, 10 mL liposomal bupivacaine into same site (*n* = 20)	Lower pain score on day of surgery No difference on postoperative days 1–5	Fewer opioid pills consumed and oral morphine equivalents on day of surgery No difference on postoperative days 1–5 No difference in total opioid pill consumption	NR	4 of 21 in Bupivacaine group (all minor) 1 of 20 in Liposomal bupivacaine group (minor)	In the liposomal bupivacaine group there was decreased pain and opioid consumption only on the day of surgery and not thereafter	Yes	1b
Davidovitch [38]	Ankle fractures (OTA/AO 44A-C) requiring ORIF	Prospective, single-blinded, randomized controlled trial	Control: local sterile saline injection (40 mL normal saline) (*n* = 39) Interventional: local liposomal bupivacaine (40 mL 1:1 of 1.3% liposomal bupivacaine and sterile saline) (*n* = 37)	Significantly lower in interventional group versus control at each time point assessed (4, 24, 48, 72, and 336 h postoperatively)	Oxycodone-aceteminophen ingestion at 4 h was less in interventional group At 48 h, the interventional group had less oxycodone-acetaminophen ingestion compared to control; however, this approached but did not reach level of statistical significance No statistically significant difference in total oxycodone-acetaminophen use over postoperative days 1–3	No difference	None	Local use of liposomal bupivacaine for ankle fractures requiring ORIF affords improved pain relief in the immediate postoperative period, resulting in a reduction in oxycodone-acetaminophen ingestion, with resultant effects seen up to 2 days postoperatively	Yes	1b
Hutchinson [39]	Periarticular femoral neck fractures treated with hemiarthoplasty	Retrospective review	Control: no local infiltration (*n* = 78) LBUP: periarticular injection of liposomal bupivacaine within a multimodal pain management program (*n* = 100)	No difference postoperative days 1–4 between control and LBUP groups	No difference in total morphine equivalents between control and LBUP groups	Significantly less in LBUP vs. control (4.8 days vs. 5.7 days)	No difference between control and LBUP groups	Supports use of local liposomal bupivacaine as part of multimodal program after hemiarthoplasty for femoral neck fractures	Yes	4
Chen [40]	Distal radius fracture with volar plating	Prospective, intervention based on surgeon preference	Control: supraclavicular nerve block alone (*n* = 20) Interventional: supraclavicular nerve block with liposomal bupivacaine (*n* = 26)	No difference in visual analog scale or QuickDASH scores between control and interventional groups at 18, 72, 168, and 336 h postoperatively	NR	NR	NR	No significant rebound pain was observed after the supraclavicular nerve block wore off following volar plating Liposomal bupivacaine did not provide measurable benefit in pain scores in patients who received a supraclavicular nerve block	No	2b
Hutchinson [41]	Fractured clavicle; subtrochanteric nonunion s/p cephalomedullary nail	Case series	20 mL liposomal bupivacaine diluted to 60 mL total volume with normal saline into platysma, pectoralis, trapezius, and deltoid at a depth of 2–3 cm using a 22 g spinal needle, additional 30 mL of 0.25% bupivacaine w/epinephrine 20 mL liposomal bupivacaine diluted to 100 mL total volume with normal saline into quadriceps, 75% anterior subfascial tissue, 25% posteriorly, additional 50 mL of 0.25% bupivacaine w/ epinephrine	NR	“occasional oral opioid” NR	NR	NR	Both patients experienced good control of postsurgical pain, supporting the clinical utility of liposomal bupivacaine in orthopedic trauma surgery	Yes	4
Herbst [42]	Talar neck fracture-dislocation with an open injury, dislocated subtalar joint, avascular talus, and considerable deformity	Case report	One vial (20 mL) of liposomal bupivacaine was mixed with 20 mL of 0.25% bupivacaine, without epinephrine and infiltrated into areas of the deep soft tissue in the peri-ankle area	At 30 h after surgery, patient reported a pain level of 0 (0–10)	NR	NR	NR	Liposomal bupivacaine use in complex foot and ankle surgery may be helpful in maintaining postoperative analgesic activity for up to 72 h	Yes	5
Amin [43]	Extracapsular and intracapsular hip fractures	Expert Panel Opinion—4 orthopedic surgeons and 3 anesthesiologists	NA	NA	NA	NA	NA	Liposomal bupivacaine should be included as part of multimodal strategies Recommend preoperative treatment with acetaminophen, NSAID, and tramadol Presurgical fascia iliaca block with bupivacaine HCl may help bridge before liposomal bupivacaine takes effect	Yes	5
Langworthy [44]	Isolated acetabular fractures	Discussion of best practices between 2 traumatologists and recommendations	NA	NA	NA	NA	NA	Liposomal bupivacaine (266 mg/20 mL) should be expanded with 50 mL of bupivacaine HCl 0.25% and saline to a total volume of 120 or 300 mL for posterior and anterior techniques, respectively 1 dose IV acetaminophen also recommended Patients also receive acetaminophen, celecoxib, gabapentin, and opioids before surgery	Yes	5

Levels of evidence were determined using the Oxford Centre for Evidence-Based Medicine: Levels of Evidence (March 2009). *NR*  result was not reported in given study. *NA*  not applicable to given study

Four studies reported a comparison between a control group and an experimental group. Different controls were used in each of these studies which consisted of a local injection of 0.5% bupivacaine without epinephrine, local saline injection, no local infiltration, or supraclavicular nerve block [37–40]. Five of the studies reported postoperative pain scores. Two studies reported significantly lower pain scores on the day of surgery (Alter and Davidovitch) [37, 38]. Alter et al. combined liposomal bupivacaine with a standard bupivacaine injection for the intervention group and compared it to a control group with a standard bupivacaine injection alone. Davidovitch et al. compared liposomal bupivacaine to a control group receiving a sterile saline injection. They also found that these lower pain scores persisted at all measured time points postoperatively [38].

Two studies compared liposomal bupivacaine to saline injection or no injection at all and found no difference in pain scores on postoperative days 1–4 [30, 32]. Chen et al. also found no difference in pain scores on postoperative days 1–4 when comparing supraclavicular nerve block with and without the addition of liposomal bupivacaine [33]. Of note, this is the only study that focused on liposomal bupivacaine’s use in a nerve block, while the other studies focused on its use as a field block. Alter et al. found that patients receiving liposomal bupivacaine had lower opioid use on the day of surgery when comparing groups of standard bupivacaine and standard bupivacaine combined with liposomal bupivacaine [30]. Hutchinson compared liposomal bupivacaine to no local injection and achieved similar pain control between groups with a decreased length of stay in the group receiving liposomal bupivacaine [39].

Three of the studies quantified narcotic consumption postoperatively by total morphine equivalents or total opioid pill consumption. These three studies (Alter, Davidovitch, Hutchinson) failed to find a significant difference between the control and study groups [37–39]. The length of stay was reported in two studies (Davidovitch, Hutchinson) and was significantly less in the liposomal bupivacaine group in one study (Davidovitch) [38, 39]. Seven of the eight studies disclosed sponsorship or funding by Pacira Pharmaceuticals, the manufacturer of liposomal bupivacaine (EXPAREL).

Three of the eight studies reported adverse events. Two of the studies found no difference between groups [38, 39]. Alter et al. found a significant difference in adverse events, all reported to be minor, which included constipation, itching, nausea, drowsiness, dizziness, and lack of energy. Alter et al. reported significantly fewer adverse events in the group treated with liposomal bupivacaine compared to local infiltration with bupivacaine. [37]

## Discussion

Although one of the original goals of this study was to quantitatively analyze the use of liposomal bupivacaine in fracture patients, this was not possible given the data available. The absence of quality data was mostly attributed to a lack of standardization of the comparator groups and outcome measurements, as well as an overall lack of data reported in the studies that were reviewed. It is commonly reported that liposomal bupivacaine has the potential to decrease pain scores, decrease length of stay, decrease opioid use, and decrease ORAEs; however, many studies only provided data on one or a few of these outcome measurements.

### Postoperative pain relief and opioid use

Another major challenge in analyzing the study results is the variation in the composition of control groups. Davidovitch et al. observed significantly lower postoperative pain scores compared to their control group; however, the control group in this study was only treated with a sham procedure, without any local anesthetic injection [38]. Liposomal bupivacaine was compared to a variety of controls in three other studies, all of which have a well-documented analgesic effect, and little difference was found in these postoperative pain scores. With these results in mind, it is difficult to suggest that liposomal bupivacaine is more effective in decreasing postoperative pain scores compared to other more commonly used analgesics. Interestingly, Alter et al. reported significantly lower pain scores on the day of surgery, and no difference thereafter, when patients were treated with liposomal bupivacaine versus a standard bupivacaine injection [37]. This finding is unexpected given that one of liposomal bupivacaine major potential benefits is an extended mechanism of action, reported to be up to 72 h.

The opportunity to decrease postoperative narcotic consumption is another major potential benefit of liposomal bupivacaine. Four of the studies reviewed in this study recorded narcotic consumption in the immediate postoperative period. Two studies demonstrated lower ingestion of narcotics in the early postoperative period. Alter et al. reported fewer opioid pills consumed on the day of surgery. Davidovitch reported oxycodone-acetaminophen ingestion at 4 h was less in the group treated with liposomal bupivacaine [37, 38]. Again, these findings are unexpected given the proposed duration of action of liposomal bupivacaine. Additionally, it is interesting that both of these studies found no difference in total narcotic consumption and narcotic consumption after postoperative day one. This was consistent across studies and all four studies reporting narcotic consumption demonstrated no difference in total morphine equivalents between groups. The lack of difference is especially notable given that in one study the control group only received a local injection of normal saline [38].

### Length of stay

Another potential therapeutic advantage of liposomal bupivacaine is its ability to relieve postoperative pain without the impaired mobilization of an indwelling catheter [12, 42]. Peripheral nerve blocks are widely used in the management of postoperative pain and may be single injection or continuous infusion through a perineural catheter [45]. While these are effective in relieving pain, single injection nerve blocks are typically limited to relieving pain for 12–24 hours [45]. Herbst suggests that in foot and ankle surgeries, in particular, there may be a role for a single injection sciatic nerve block with liposomal bupivacaine alone which could potentially provide pain relief for 72 h and shorten length of stay [42]. Despite this purported benefit, only two studies compared the length of stay between control and experimental groups. Notably, Davidovitch et al. found no difference in length of stay between patients treated with local injection of normal saline and patients treated with local injection of liposomal bupivacaine [38]. In contrast, Hutchinson found that patients treated with liposomal bupivacaine versus no local injection spent significantly less time in the hospital with an average of 4.8 days and 5.7 days, respectively [39].

It is impossible to confidently determine whether the results of Davidovitch et al. or Hutchinson are more typical in a given clinical scenario. Notably, the study population differed in that Davidovitch studied ankle fractures requiring open reduction and internal fixation (ORIF), while Hutchinson studied periarticular femoral neck fractures treated with hemiarthroplasty. Many experts have reported that the method of expanding liposomal bupivacaine, injection technique, and location are critical to success and this may have also a role in the differing results of these studies [43, 44, 46]. Regardless of the origin of the conflicting results, it is clear that additional studies—focused on both ankle fractures and femoral neck fractures—will be beneficial in elucidating the role of liposomal bupivacaine in postoperative pain management and shortening average length of stay.

### Financial impact

Calculating the financial impact of introducing a new drug to a multimodal treatment regimen is difficult. Both the direct costs of the drug and the indirect costs of the implementing the new drug must be taken into account. Multiple studies have reported an institutional cost of liposomal bupivacaine of approximately $315 [47, 48]. A standard bupivacaine or similar local anesthetic cocktail costs substantially less and are reported to range from $4.92 to $27 per dose [47, 48]. The indirect costs of implementing a new drug are more difficult to account for; however, proxies such as length of stay, overall opioid use, and need for intensive care unit monitoring have been used to help approximate these cost changes [36].

None of the studies identified in this investigation reported on the overall difference in health care costs between the control group and the experimental group. This is another area that should be investigated by future studies focused on fracture patients. In patients undergoing total knee arthroplasty or total hip arthroplasty, many studies have found that the use of liposomal bupivacaine significantly reduces total hospital costs [46, 49, 50]. This reduction in hospital costs is mostly attributed to shorter length of stays and earlier initiation of rehabilitation [49]. If there are similar cost-savings in fracture patients, this could be important, given the increasing pressure to deliver value-based care using evidence-based practices.

Of the eight studies included in the final qualitative analysis of this study, seven reported sponsorship or a conflict of interest with Pacira Pharmaceuticals. Notably, these studies also tended to present favorable study conclusions on the use of liposomal bupivacaine in the given patient population. While the underlying biases in these studies should not nullify the overall conclusions, there is an obvious need for prospective, randomized trials that are non-biased and not sponsored by the pharmaceutical industry. Ideally, these studies may also focus on procedures that can be performed bilaterally such as bunion surgery or total knee arthroplasty. In this way, the use of liposomal bupivacaine can be directly compared to the use of a standard local anesthetic, such as lidocaine or bupivacaine, in the contralateral side. Using this study design, a randomized, double-blind prospective trial could nearly eliminate the variation and confounding factors seen in earlier studies, further clarifying the effect of liposomal bupivacaine. Until then, clinicians should continue to maintain a healthy skepticism and rely on their own interpretation of the reported data, prior to widely implementing the use of liposomal bupivacaine based on current conclusions [22].

### Safety

Three studies included in this study reported adverse events. Of these, only one study, Alter et al., reported a significant difference in adverse event rate between the control and experimental groups [37]. There were a total of five individuals who reported adverse events, with only one patient suffering an adverse event in the group treated with liposomal bupivacaine. All of these events were reported to be minor and included constipation, itching, nausea, drowsiness, dizziness, and lack of energy.

Another commonly cited concern of local anesthetic injections are the potential for cardiac toxicity (arrest) and neurotoxicity (seizures) [51]. There are also some reports of myotoxicity associated with bupivacaine and ropivacaine [52]. Further, it is logical that with new delayed formulations of local anesthetics, particularly bupivacaine, there may be an increased the risk for such toxicities. Many studies have evaluated the safety of liposomal bupivacaine in various settings, including local injection into soft tissues and periarticular injections, and demonstrated a favorable side effect profile with few serious adverse events [52–55]. Of note, Soberón et al. published a case report documenting compartment syndrome in a patient treated with perineural liposomal bupivacaine and note the potential for a delayed diagnosis in similar patients [56].

### Conclusions

In conclusion, there is currently a lack of evidence to support or oppose the use of liposomal bupivacaine in fracture patients. Prospective, randomized clinical trials should be conducted in this patient population without funding from the pharmaceutical industry. These studies will be most effective if they compare the use of liposomal bupivacaine to the current standard of care for postoperative pain management and are incorporated as part of a multimodal approach to analgesia. Additionally, these studies should analyze many variables and at a minimum record data on the potential benefits of liposomal bupivacaine. These variables include postoperative pain scores, total narcotic consumption, length of stay, adverse events, and total hospitalization cost. There is also significant potential to further evaluate the injection technique used during the administration of liposomal bupivacaine in order to create a more standardized procedure which may contribute to more reliable results. Specifically, this may involve studying if the efficacy of liposomal bupivacaine changes based on how and where it is administered. For example, studies may compare administering liposomal bupivacaine directly into soft tissues versus intra-articular injection versus as an adjunct to peripheral nerve blocks. Throughout these studies, the safety of liposomal bupivacaine should continue to be monitored to ensure there are no detrimental effects that have not been previously identified.

## Data Availability

All literature cited in this manuscript is available online or in print through scholarly journals. The data were compiled into a table which is included in the manuscript for ease of reference.
